# Deep Hypothermic Circulatory Arrest Does Not Show Better Protection for Vital Organs Compared with Moderate Hypothermic Circulatory Arrest in Pig Model

**DOI:** 10.1155/2019/1420216

**Published:** 2019-04-17

**Authors:** Yang Liu, Zining Wu, Lu Dai, Haiyang Li, Ming Gong, Feng Lan, Xinliang Guan, Hongjia Zhang

**Affiliations:** Department of Cardiac Surgery, Beijing Anzhen Hospital, Capital Medical University, Beijing Institute of Heart, Lung and Blood Vessel Diseases, Beijing Lab for Cardiovascular Precision Medicine, Beijing Aortic Disease Center, Cardiovascular Surgery Center, Beijing Engineering Research Center for Vascular Prostheses, Beijing, China

## Abstract

**Background:**

Continued debates exist regarding the optimal temperature during hypothermic circulatory arrest in aortic arch repair for patients with type A aortic dissection. This study seeks to examine whether the use of moderate hypothermic circulatory arrest in a pig model provides comparable vital organ protection outcomes to the use of deep hypothermic circulatory arrest.

**Methods:**

Thirteen pigs were randomly assigned to 30 minutes of hypothermic circulatory arrest without cerebral perfusion at 15°C (n = 5), 25°C (n = 5), and a control group (n = 3). The changes in standard laboratory tests and capacity for protection against apoptosis in different vital organs were monitored with different temperatures of hypothermic circulatory arrest management in pig model to determine which temperature was optimal for hypothermic circulatory arrest.

**Results:**

There were no significant differences in the capacity for protection against apoptosis in vital organs between 2 groups (p > 0.05, respectively). Compared with the moderate hypothermic circulatory arrest group, the deep hypothermic circulatory arrest group had no significant advantages in terms of the biologic parameters of any other organs (p > 0.05).

**Conclusions:**

Compared with deep hypothermic circulatory arrest, moderate hypothermic circulatory arrest is a moderate technique that has similar advantages with regard to the levels of biomarkers of injury and capacity for protection against apoptosis in vital organs.

## 1. Introduction

Acute aortic dissection (AAD) is a serious and rapidly progressing vascular disease that has features of acute onset and poor prognosis. In China, the incidence of the disease is increasing every year, and new cases occur in more than 100 thousand people every year, which is far more than those in Western countries [1, 2]. At present, the most effective treatment is still a surgical procedure, that is, the use of artificial grafts to replace the diseased vessels; however, the operation, which has high complication and mortality rates, is vulnerable to multiple organ injuries [3, 4]. Currently, surgical operation under moderate to deep hypothermic circulatory arrest (DHCA) is the most effective treatment for this disease to avoid or reduce the ischemia and anoxia of vital organs caused by circulatory arrest. However, with the increase in clinical experience, people have gradually realized that the deep hypothermic circulation also has many disadvantages and is usually associated with potential side effects. At present, many scholars have abandoned traditional DHCA and adopted moderate hypothermic circulatory arrest (MHCA) or even mild hypothermic circulatory arrest combined with cerebral perfusion, but these attempts lack a foundation [5, 6]. Due to the limitations of clinical research, some fundamental research is urgently needed for the detailed and in-depth study of the pathophysiology caused by hypothermic circulatory arrest (HCA). Currently, there is no common guideline on what temperature should be adopted for HCA with or without selective cerebral perfusion.

The aim of this study is to compare the standard laboratory tests and capacity for protection against apoptosis in different vital organs in a pig model using DHCA with those of a matched group of pigs using MHCA.

## 2. Methods

### 2.1. Study Design

Thirteen pigs (Animal Experiment Center of Fuwai Hospital, Beijing) of either sex, between 4 and 5 months old and weighing 100 ± 5.1 kg, were randomly assigned to 30 minutes of HCA without cerebral perfusion at 15°C (n = 5), 25°C (n = 5), and a control group (n = 3). All the operations on experimental animals were examined by the Ethical Association of Animal Experimentation. The experimental animals were anesthetized with 1% isoflurane inhaled anesthesia and intravenous administration of 6 mg/kg/h propofol and 2 *μ*g/kg fentanyl, intubated, and mechanically ventilated with a volume-cycled respirator. Anesthesia was maintained with IV infusion of fentanyl, suxamethonium chloride, and propofol. An arterial pressure catheter was inserted via the Era-artery for pressure monitoring and blood gas analysis, and a venous catheter was inserted via the right femoral vein for fluid administration and blood sampling. The nasopharyngeal temperature was monitored by imbedding a temperature probe. The hemodynamic variables that were monitored consisted of blood pressure, heart rate, oxygen partial pressure, and electrocardiogram (ECG).

### 2.2. Operation Management

**Figure 1:**
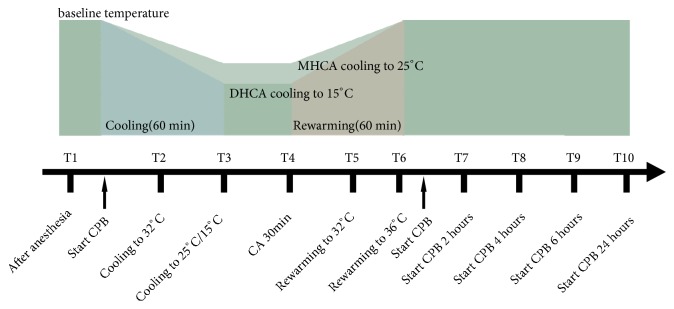
Experiment procedures. The pigs underwent 60 min of cooling, 30 min of deep hypothermic circulatory arrest (DHCA)/moderate hypothermic circulatory arrest (MHCA), and 60 min of rewarming. After stopping CPB, the experiment continued for 24 hours. CPB = cardiopulmonary bypass; CA = cardiac arrest.

The extracorporeal circuit was primed with Gelofusine and sodium lactate Ringer's injection. The hematocrit was 20-24% after dilution in cardiopulmonary bypass (CPB). During complete cardiopulmonary bypass, the flow was maintained at 90-100 ml/kg/min, and the average arterial pressure was maintained at 60-90 mmHg. *α*-stat could be used to maintain the homeostasis and avoid the deficiency of arterial oxygen partial pressure. Both cooling and rewarming were performed by a heat exchanger. In the DHCA group, pigs were cooled for approximately 60 minutes. When the nasopharyngeal temperature reached 15°C, the heat exchanger continued to run and maintained the nasopharyngeal temperature at 15°C, and CPB was stopped. After 30 minutes of hypothermic circulatory arrest, CPB was continued, and the pigs were rewarmed to the nasopharyngeal temperature of 36°C. When hemodynamically stable, the pigs were weaned from CPB and continued to survive for 24 hours. In the MHCA group, the establishment of CPB and the operation procedure were the same as those in the DHCA group, but the target temperature was 25°C. No procedures were performed in the control group. The experimental procedures were shown in Figure 1.

### 2.3. Time Points

There were ten time points in this experiment: after anesthesia; cooling to 32°C; cooling to target temperature; cardiac arrest (CA) 30 min; rewarming to 32°C; rewarming to 36°C; and 2 hours, 4 hours, 6 hours, and 24 hours after weaning from CPB (Figure 1).

### 2.4. Blood Sample and Tissue Harvesting

Blood samples were collected at every time point in the experiment, as illustrated in Figure 1. Blood samples (20 ml) were taken from the right femoral artery. After the experiment, the pigs were euthanized and dissected under anesthesia. The tissues of vital organs, such as brain, lung, liver, and kidney, were then harvested. The creatinine (Cr), blood urea nitrogen (BUN), alanine aminotransferase (ALT), and aspartate aminotransferase (AST) were tested from the blood samples. Transferase-mediated deoxyuridine triphosphate-biotin nick end labeling (TUNEL) technology was used to stain the specimens to show the apoptotic cells in the vital organs and calculate the apoptosis index (%). The apoptosis index = (apoptotic cells/total cells) × 100%. Immunohistochemistry methods were used to detect the expression and distribution of Bax, Bcl-2, and caspase-3, which were all apoptosis-related proteins in the specimens. The positive cell rate (%) was calculated to indicate the capacity for protection against apoptosis in vital organs.

### 2.5. Statistical Analysis

The Kolmogorov-Smirnov method was used to detect the normality of data distribution. The measurement data were the mean number of standard deviation (SD), and the single factor analysis of variance was adopted in the group. The number of digits (four division spacing) indicated that the Wilcoxon rank sum test was used for the comparison between groups. Count data used percentages or rate tables. In comparison, the Chi square test or Fisher test was used. The t test was used for comparison between the 10 time points. Repeated measurements were analyzed with Bonferroni correction analysis. All statistical analyses used SPSS 18.0 statistical software and a bilateral test, and p < 0.05 was considered statistically significant.

## 3. Results

### 3.1. The Capacity for Protection against Apoptosis

#### 3.1.1. The Capacity for Protection against Apoptosis in the Brain

**Figure 2:**
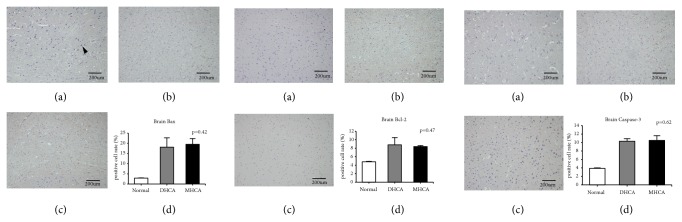
The positive cell rate of Bax, Bcl-2, and caspase-3 in the normal group (a), DHCA group (b), and MHCA group (c). (d) showed the comparison of the positive cell rate among the three groups (p = 0.42, p = 0.47, and p = 0.62, respectively).

**Figure 3:**
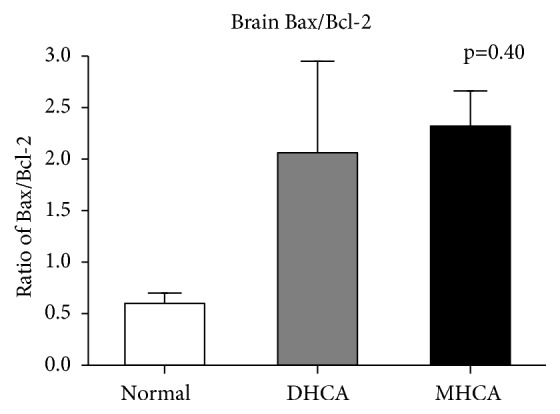
The comparison of Bax/Bcl-2 among the three groups (p = 0.40).

**Figure 4:**
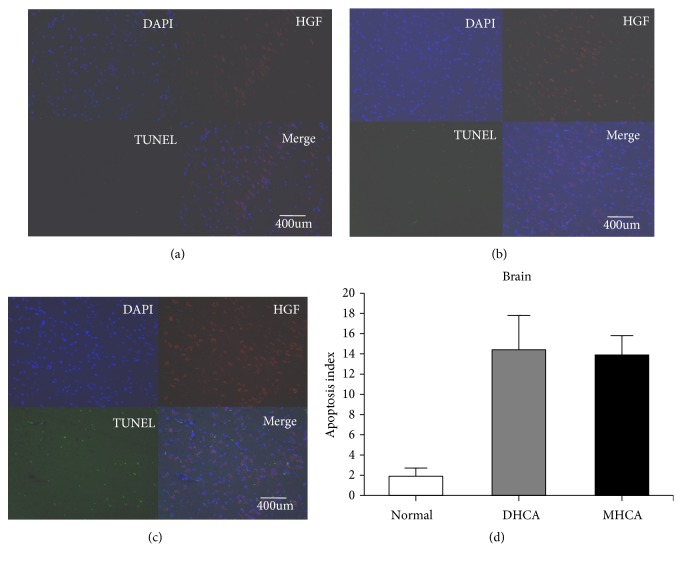
The apoptosis index in the normal group (a), MHCA group (b), and DHCA group (c). (d) showed the comparison of the apoptosis index among the three groups (p = 0.69).

As shown in Figures 2, 3, and 4, for the brain, there were no significant differences in the apoptosis index and positive cell rate of Bax, Bcl-2, and caspase-3 between the MHCA group and DHCA group, and there was no difference in Bax/Bcl-2 either, which indicated a similar capacity for protection against apoptosis in the brain under different HCA conditions (p > 0.05, respectively) (Figures 2, 3, and 4).

#### 3.1.2. The Capacity for Protection against Apoptosis in the Lung

**Figure 5:**
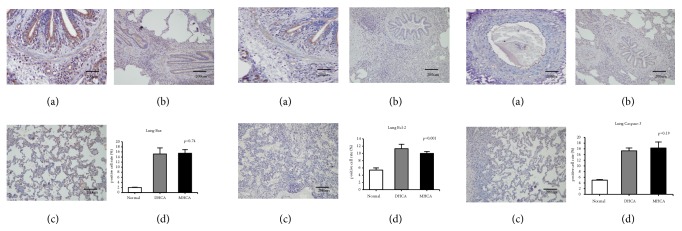
The positive cell rate of Bax, Bcl-2, and caspase-3 in the normal group (a), DHCA group (b), and MHCA group (c). (d) showed the comparison of the positive cell rate among the three groups (p = 0.74, p = 0.001, and p = 0.19, respectively).

**Figure 6:**
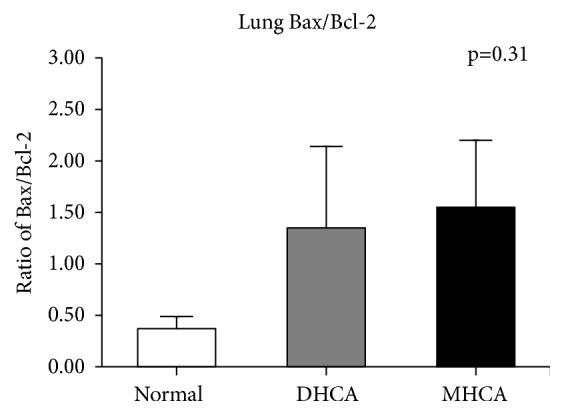
The comparison of Bax/Bcl-2 among the three groups (p = 0.31).

**Figure 7:**
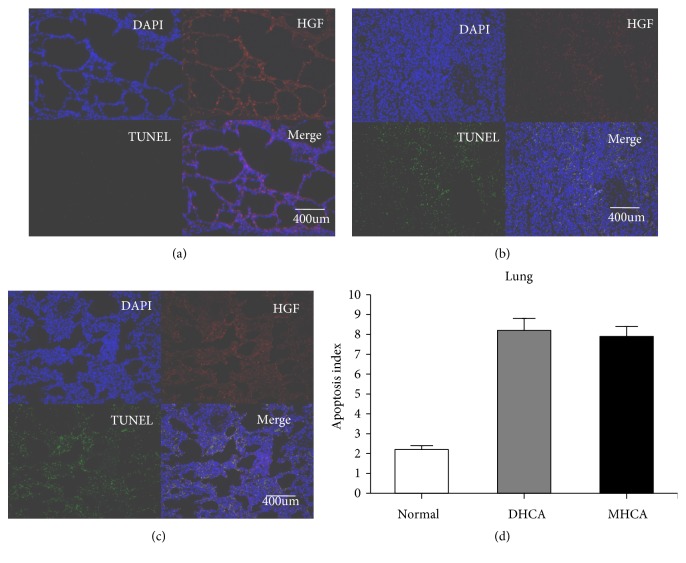
The apoptosis index in the normal group (a), MHCA group (b), and DHCA group (c). (d) showed the comparison of the apoptosis index among the three groups (p =0.24).

As shown in Figures 5, 6, and 7, for the lung, there was a significant difference in Bcl-2 between the MHCA group and DHCA group. However, there were no significant differences in the apoptosis index and positive cell rate of Bax, caspase-3, and Bax/Bcl-2, which still indicated a similar capacity for protection against apoptosis in the lung under different HCA conditions (Figures 5, 6, and 7).

#### 3.1.3. The Capacity for Protection against Apoptosis in the Liver

**Figure 8:**
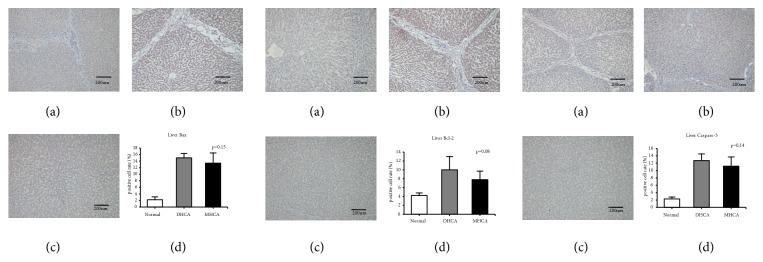
The positive cell rate of Bax, Bcl-2, and caspase-3 in the normal group (a), DHCA group (b), and MHCA group (c). (d) showed the comparison of the positive cell rate among the three groups (p = 0.15, p= 0.08, and p = 0.14, respectively).

**Figure 9:**
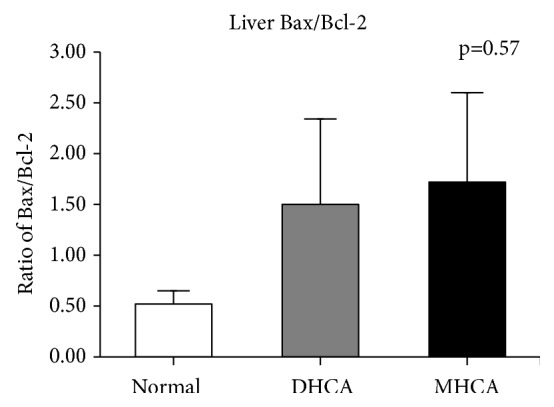
The comparison of Bax/Bcl-2 among the three groups (p = 0.57).

**Figure 10:**
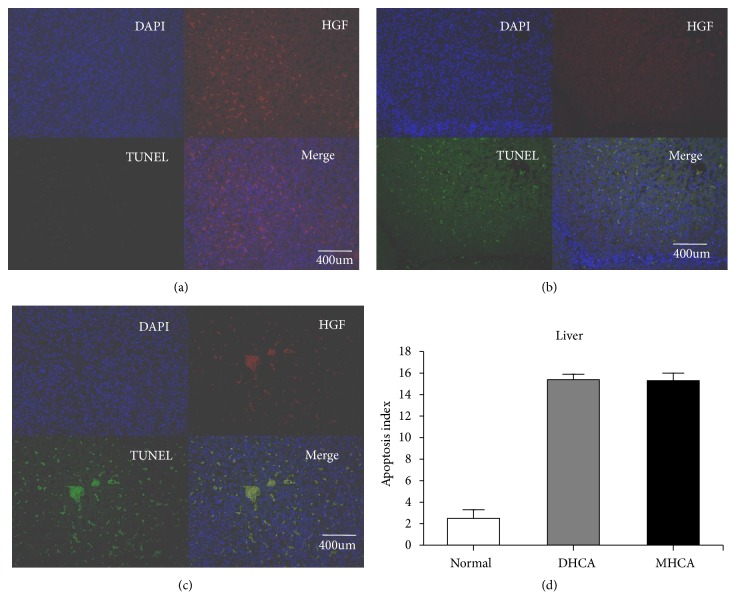
The apoptosis index in the normal group (a), MHCA group (b), and DHCA group (c). (d) showed the comparison of the apoptosis index among the three groups (p =0.72).

As shown in Figures 8, 9, and 10, for the liver, there were no significant differences in the apoptosis index and positive cell rate of Bax, Bcl-2, and caspase-3 between the MHCA group and DHCA group, and there was no difference in Bax/Bcl-2, which indicated that the capacity for protection against apoptosis in the liver under different HCA conditions was the same (p > 0.05, respectively) (Figures 8, 9, and 10).

#### 3.1.4. The Capacity for Protection against Apoptosis in the Kidney

**Figure 11:**
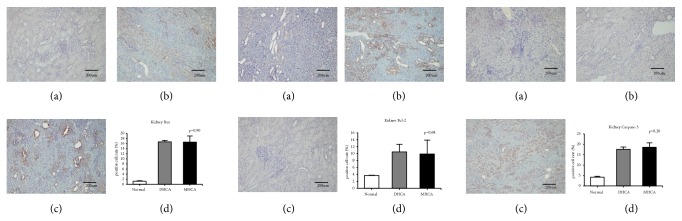
The positive cell rate of Bax, Bcl-2, and caspase-3 in the normal group (a), DHCA group (b), and MHCA group (c). (d) showed the comparison of the positive cell rate among the three groups (p = 0.90, p= 0.68, and p = 0.20, respectively).

**Figure 12:**
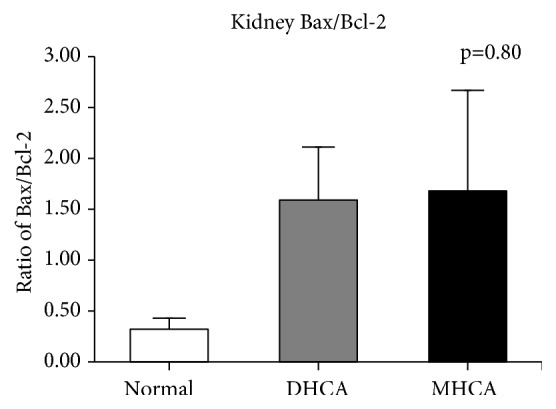
The comparison of Bax/Bcl-2 among the three groups (p = 0.80).

**Figure 13:**
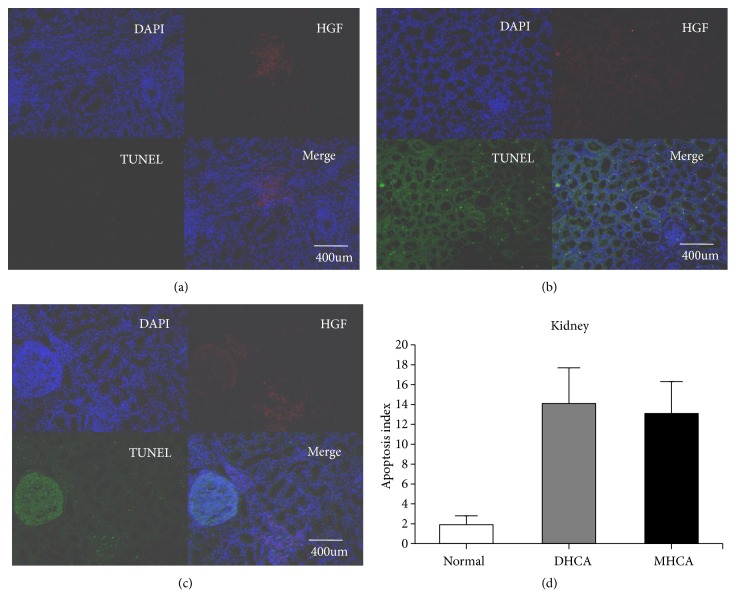
The apoptosis index in the normal group (a), MHCA group (b), and DHCA group (c). (d) showed the comparison of the apoptosis index among the three groups (p =0.52).

As shown in Figures 11, 12, and 13, for the kidney, there were no significant differences in the apoptosis index and positive cell rate of Bax, Bcl-2, and caspase-3 between the MHCA group and DHCA group, and there was no difference in Bax/Bcl-2, which indicated that the capacity for protection against apoptosis in the kidney under different HCA conditions was the same (p > 0.05, respectively) (Figures 11, 12, and 13).

### 3.2. Standard Laboratory Tests

**Figure 14:**
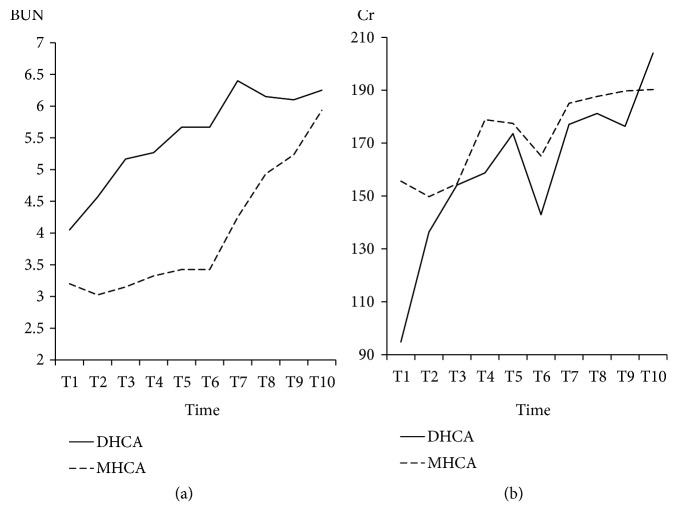
The blood urea nitrogen (BUN) and creatinine (Cr). (a) Blood urea nitrogen; (b) creatinine; p < 0.05, deep hypothermic circulatory arrest (DHCA) versus moderate hypothermic circulatory arrest (MHCA).

The BUN and Cr data were shown in Figures 14(a) and 14(b). BUN seemed to be higher in the DHCA group than that in the MHCA group at all time points, but this difference was not statistically significant. Cr was similar between the two groups, and there was no significant difference at all time points (p > 0.05, respectively) (Figure 14).

**Figure 15:**
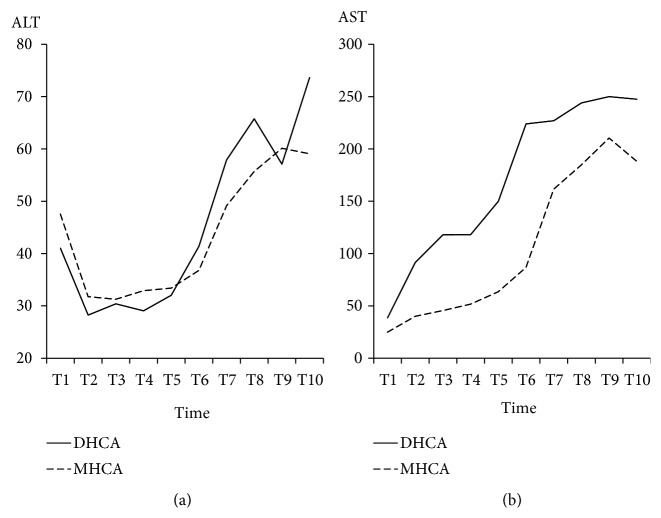
The alanine aminotransferase (ALT) and aspartate aminotransferase (AST). (a) Alanine aminotransferase; (b) aspartate aminotransferase; p < 0.05, deep hypothermic circulatory arrest (DHCA) versus moderate hypothermic circulatory arrest (MHCA).

The ALT and AST data were given in Figures 15(a) and 15(b). For ALT, there was no significant difference at all time points. AST seemed higher in the DHCA group than that in the MHCA group at all time points, but this difference was not statistically significant (p > 0.05, respectively) (Figure 15).

## 4. Discussion

Surgical treatment under HCA is currently the main means of treating complicated congenital heart disease and disease involving the aortic arch, mainly including type A aortic dissection. According to the following criteria, hypothermia is divided into three groups: mild hypothermia 28.1-34°C, moderate hypothermia 20.1-28°C, deep hypothermia 14.1-20°C, and profound hypothermia ≤ 14°C [7]. In the past, DHCA was generally considered a safe and effective method; however, in clinical practice, it was gradually found that hypothermia, especially deep hypothermia, was associated with many adverse effects on patients, particularly the injury of blood coagulation function, which led to wound and anastomotic bleeding in patients [8, 9]. Some previous studies have confirmed that blood loss is the main cause of early postoperative death in patients with aortic dissection [5, 10]. The previous clinical research of our team has also reflected this fact. Guan et al. [11] studied 62 patients who underwent emergent aortic arch surgery for Stanford type A acute aortic dissection and collected blood samples at different time points in the perioperative period. It was found that HCA led to disorders of blood coagulation function and significantly decreased the function of blood coagulation factors and platelets. As the temperature decreased, the blood coagulation system suffered more injuries, and more complications occurred. Keenan and his colleagues [12] studied 571 patients who underwent hemiarch replacement, including DHCA and MHCA, and found that MHCA slightly reduced perioperative blood loss and plasma transfusion requirements compared with DHCA. In addition, the long cooling and rewarming process prolonged the time of CPB, which was also an important reason for the damage of various systems. DHCA could also cause fluid retention and affect postoperative respiratory function [4]. Tirilomis et al. [13] analyzed the damage of liver tissue in neonatal piglets after surgery under CPB and DHCA. After the surgery, the liver tissue biopsy was taken, and the state of inflammatory reaction and liver tissue edema were analyzed. The results showed that DHCA decreased (or at least slowed) the inflammatory reaction but increased the edema of hepatic cells. Cooper et al. [14] also found that endothelial dysfunction in cerebral microvessels, large-caliber renal arteries, and pulmonary veins was associated with DHCA. DHCA was also associated with duodenal apoptosis. In other words, these adverse factors have significantly increased the risk of surgery.

In recent years, with the continuous improvement of the level of surgical techniques and materials and the application of various selective cerebral perfusion techniques, the degree of ischemia and anoxia in vital organs of the patients has been reduced effectively. Additionally, the range of hypothermia during surgery could be improved, and the blood coagulation dysfunction as well as the tissue damage caused by DHCA could be reduced. Clinically, it is difficult to obtain visceral specimens directly after surgery. The degree of organ injuries can only be indirectly understood by the biologic parameters in the blood samples. In our study, pigs were used to establish DHCA models, which were similar to human genetic characteristics. The visceral specimens were collected directly after the surgery, and the pathological analysis of the visceral specimens was performed. At the same time, combined with the biologic parameters of vital organs between two groups, the differences in vital organ injuries without cerebral perfusion were studied systematically and integrally. This approach provides a theoretical basis for the selection of hypothermic strategy in the treatment of type A aortic dissection.

The research on the nervous system after HCA has been a hot topic. However, there are few studies on other vital organs, such as the lung, liver, and kidney. Currently, more scholars have begun to pay attention to the protection of vital organs other than the nervous system. We analyzed the degree of injuries of all vital organs in the experimental animals after HCA. A comprehensive and intuitive description of vital organ protection was given. We stained the visceral specimens by TUNEL, which showed the number of apoptotic cells in each organ, and we calculated and analyzed the apoptosis index of each organ. We also analyzed the positive cell rate of each organ in the proteins that were related to apoptosis, such as Bax, Bcl-2, and caspase-3, by an immunohistochemical technique. The capacity for protection against apoptosis in vital organs could be measured by these proteins. Bax is a water-soluble protein that is homologous to Bcl-2. The Bax gene is the apoptosis promoting gene in the Bcl-2 gene family. The overexpression of Bax can antagonize the protective effect of Bcl-2 and cause the cell to die, and the Bcl-2 protein can inhibit cell death caused by many cytotoxic factors [15, 16]. It was found that the ratio between Bax and Bcl-2 was the critical factor determining the inhibition of apoptosis. The traditional theory holds that deep hypothermia is better than moderate hypothermia for vital organ protection, but our results showed that there were no significant differences, except for Bcl-2 protein in the brain, which meant that the injuries of vital organs were the same between two groups. This result was different from that of the traditional theory. Some scholars have also drawn different conclusions through clinical research that are different from those of traditional theories. Keeling et al. [17] reviewed 3,265 patients undergoing total aortic arch replacement using antegrade cerebral perfusion, and it was found that there was no difference in neurologic outcomes or in-hospital mortality between MHCA and DHCA groups. Gong [18] on our team reached a similar conclusion in his article. Arnaoutakis and his colleagues [19] found that, compared with DHCA/RCP, an MHCA/ACP cerebral protection strategy did not appear to be associated with worse postoperative renal outcomes. Liang et al. [20] had obtained similar conclusions through animal experiments. These authors found that moderate hypothermia was less detrimental to the intestinal barrier than deep hypothermia. In fact, the results of these studies show that hypothermia does not bring much benefit to the protection of vital organs. Sometimes, this treatment can be a double-edged sword that also has many adverse effects. In this situation, we begin to reflect on whether the use of “deep hypothermia” is appropriate. In recent years, these studies have also prompted us that proper temperature increase does not cause more injuries to vital organs and sometimes can even bring some benefits.

We also analyzed the biologic parameters of vital organs, including Cr, BUN, ALT, and AST, which were associated with renal and liver function. The results showed that there was no significant difference between two groups. This finding further demonstrated our conclusion that there was no significant difference in the protection of vital organs between the DHCA and MHCA groups without cerebral perfusion. Recently, some other studies have shown that postoperative systemic inflammatory response syndrome (SIRS) is the pathophysiological basis of vital organ dysfunction, and it can eventually develop into multiple organ dysfunction syndrome (MODS) [21, 22]. Heart failure, respiratory failure, hemodynamic disorders, and liver and kidney dysfunction after CPB are directly or indirectly related to SIRS. Oxidative stress is an important mechanism of ischemia-reperfusion injury. During oxidative stress, a large number of oxygen free radicals attack polyunsaturated fatty acids in biofilm phospholipids, causing membrane lipid peroxidation, swelling, dissolution of mitochondria, polyribosome depolymerization, abscission, and leakage of lysosomes resulting in cell dysfunction. Therefore, in future animal experiments, the protection of vital organs after surgery can be further evaluated by observing the levels of superoxide dismutase (SOD) and malonaldehyde (MDA), inflammatory cells, and inflammatory factors [23]. In conclusion, MHCA has less cooling and rewarming time, which can reduce the injuries caused by long-time cardiopulmonary bypass to vital organs. Furthermore, the influence of MHCA on the blood coagulation system is less than that of DHCA. In the future, we have no reason to doubt that there will be more surgeries under MHCA to avoid the adverse effects of deep hypothermia.

This article also has some limitations. Limited by the cost of large animal experiments, we set the temperature of deep hypothermia to 15°C and the temperature of moderate hypothermia to 25°C. We have not done any further research on other temperatures in these two groups. Therefore, in future animal experiments, more groups will be studied. Our previous clinical study also showed the effect of deep hypothermia on the blood coagulation system; however, in this animal experiment, we did not perform similar studies. The effect of deep hypothermia on the blood coagulation system is also a hot topic that is both attention-grabbing and controversial. Further fundamental research is needed. In addition, surgery utilizing HCA is currently often combined with cerebral perfusion. Under this circumstance, whether the protection of vital organs in the two groups still has the abovementioned conclusion requires more animal experiments for justification.

## 5. Conclusion

Compared with DHCA, MHCA is independently associated with an equivalent role in the protection of vital organs. Our results suggest that vital organs can be well protected by MHCA. This study implies the clinical safety and efficacy of MHCA in emergency aortic arch repair, and MHCA can provide equivalent protection of vital organs without increasing the risk of operative mortality and morbidity.

## Data Availability

The data used to support the findings of this study are available from the corresponding author upon request.

## Abbreviations

AAD: Acute aortic dissection
ALT: Alanine aminotransferase
AST: Aspartate aminotransferase
BUN: Blood urea nitrogen
CA: Cardiac arrest
CPB: Cardiopulmonary bypass
Cr: Creatinine
DHCA: Deep hypothermic circulatory arrest
ECG: Electrocardiogram
HCA: Hypothermic circulatory arrest
MDA: Malonaldehyde
MHCA: Moderate hypothermic circulatory arrest
MODS: Multiple organ dysfunction syndrome
SD: Standard deviation
SIRS: Systemic inflammatory response syndrome
SOD: Superoxide dismutase
TUNEL: Transferase-mediated deoxyuridine triphosphate-biotin nick end labeling.
